# The Two Editors

**Published:** 1882-07

**Authors:** J. B. Hodgkin


*The Two Editors.*—The undersigned has of late, for just reasons, taken the trouble to sign his name to editorial articles written by him. Opinions and sentiments the reverse of those entertained by him are entertained and expressed by Dr. Gorgas. This he has a perfect right to do; but it is unfair to credit the associate editor with them, or, on the other hand, to ascribe to Dr. Gorgas the ideas the writer may print. It will be understood therefore—this seems *not* to be universally understood—that *unsigned* editorial articles are not those of

J. B. HODGKIN.

